# Misinformation, chiropractic, and the COVID-19 pandemic

**DOI:** 10.1186/s12998-020-00353-2

**Published:** 2020-11-18

**Authors:** Iben Axén, Cecilia Bergström, Marc Bronson, Pierre Côté, Casper Glissmann Nim, Guillaume Goncalves, Jeffrey J. Hébert, Joakim Axel Hertel, Stanley Innes, Ole Kristoffer Larsen, Anne-Laure Meyer, Søren O’Neill, Stephen M. Perle, Kenneth A. Weber, Kenneth J. Young, Charlotte Leboeuf-Yde

**Affiliations:** 1 Karolinska Institutet, Institute of Environmental Medicine, Unit of Intervention and Implementation Research for worker health, Stockholm, Sweden; 2 The Norwegian Chiropractic Research Foundation “Et liv I Bevegelse”, ELIB, Oslo, Norway; 3 Umeå University, Department of Clinical Sciences, Unit of Obstetrics and Gynecology, Umeå, Sweden; 4 Evidence Based Chiropractic Network, Private practice, Kirkland Lake, Ontario Canada; 5 Faculty of Health Sciences, Ontario Tech University and Centre for Disability Prevention and Rehabilitation at Ontario Tech University and CMCC, Oshawa, Ontario Canada; 6 Spinecentre of Southern Denmark, University Hospital of Southern Denmark, Odense, Denmark; 7 Institute of Regional Health Research, University of Southern Denmark, Odense, Denmark; 8 Institut Franco-Européen de Chiropraxie, Ivry-sur-Seine, France; 9 Faculty of Kinesiology, University of New Brunswick, Fredericton, Canada; 10 Discipline of Psychology, Exercise Science, Counselling and Chiropractic, Murdoch University, Perth, Australia; 11 Private practice, Tønsberg, Norway; 12 College of Health Sciences, School of Chiropractic, University of Bridgeport, Bridgeport, CT USA; 13 Stanford University School of Medicine, Department of Anesthesiology, Perioperative and Pain Medicine, Division of Pain Medicine, Systems Neuroscience and Pain Lab, Palo Alto, California, USA; 14 University of Central Lancashire, School of Sport and Health Sciences, Preston, UK

**Keywords:** Covid-19, Chiropractors, Spinal manipulation, Professional traditionalism, Infodemic, Debate

## Abstract

**Background:**

In March 2020, the World Health Organization elevated the coronavirus disease (COVID-19) epidemic to a pandemic and called for urgent and aggressive action worldwide. Public health experts have communicated clear and emphatic strategies to prevent the spread of COVID-19. Hygiene rules and social distancing practices have been implemented by entire populations, including ‘stay-at-home’ orders in many countries. The long-term health and economic consequences of the COVID-19 pandemic are not yet known.

**Main text:**

During this time of crisis, some chiropractors made claims on social media that chiropractic treatment can prevent or impact COVID-19. The rationale for these claims is that spinal manipulation can impact the nervous system and thus improve immunity. These beliefs often stem from nineteenth-century chiropractic concepts. We are aware of no clinically relevant scientific evidence to support such statements.

We explored the internet and social media to collect examples of misinformation from Europe, North America, Australia and New Zealand regarding the impact of chiropractic treatment on immune function. We discuss the potential harm resulting from these claims and explore the role of chiropractors, teaching institutions, accrediting agencies, and legislative bodies.

**Conclusions:**

Members of the chiropractic profession share a collective responsibility to act in the best interests of patients and public health. We hope that all chiropractic stakeholders will view the COVID-19 pandemic as a call to action to eliminate the unethical and potentially dangerous claims made by chiropractors who practise outside the boundaries of scientific evidence.

## Background

In March 2020, the World Health Organisation (WHO) labelled the outbreak of *severe acute respiratory syndrome coronavirus 2* (SARS-CoV-2), COVID − 19, a pandemic and called for countries to take urgent and aggressive action [1]. Because there is no known treatment for this virus, we are being asked to observe social distancing, wash our hands frequently, and curtail our activities with those outside our household. Most businesses that include person-to-person contact have been closed.

Despite this grave situation, some chiropractors have advocated a misbelief that spinal manipulative therapy (SMT) or “adjustments” can boost immunity and thus should be offered as a preventive measure for viral infections. The World Federation of Chiropractic (WFC) noted this development on March 17th 2020 and refuted this in a public statement: “… .there is no credible scientific evidence to support this notion and to suggest otherwise is potentially dangerous to public health” [2]. Nevertheless, some chiropractors continue promoting misinformation on social media putting the chiropractic profession at odds with scientific evidence.

Evidence-based chiropractic care provides management of musculoskeletal disorders, offering an array of clinical services for a limited range of conditions [3, 4]. The literature demonstrates the deleterious impact of musculoskeletal disorders world-wide [5–7]. The evidence based approach to the delivery of chiropractic services has made it possible for the chiropractic profession to contribute to health care as an accountable profession, rooted in science, increasingly steeped in academia, and continuously seeking to improve the efficacy and safety of the clinical services it provides [4–6]. This is precisely the role ascribed to chiropractors by the general public [8]. The relevance of the chiropractic profession in this context is not in question.

However, some “traditional”(according to the Oxford dictionary: “being part of the beliefs, customs or way of life of a particular group of people, that have not changed for a long time” [9]) chiropractors uphold the historical belief that adjustments can correct spinal lesions (*subluxations*) [10, 11], responsible for almost all disease [12], and that adjustments will improve the brain-body-environment communication [13]. Recent studies suggest that approximately 20% of chiropractors have this focus [14, 15]. Many of their claims relate to relatively benign conditions [16, 17] or ones where a musculoskeletal origin is at least possible [18, 19].

The issue of making false claims by chiropractors has previously been raised as a matter of concern both from outside the profession [20] and from within [21]. While inappropriate claims made by “traditional” chiropractors are never in the public’s interest, the specific claims of boosting immunity during the COVID-19 crisis presents a fundamentally different level of risk of harm to patients and public health. Misinformation about adjustments and immunity taints public understanding of viral prevention, undermines the coordinated efforts of health authorities, and has become a cause for concern among researchers and public health authorities [22, 23].

Statements related to the COVID-19 pandemic demonstrate that the chiropractic profession includes two distinct groups that have little in common and that mainstream chiropractors can no longer accept and protect the “traditional” fringe, because it presents a danger to the public [24].

Thus, the purpose of our article is to explore scientifically unsubstantiated statements by “traditional” chiropractors to stimulate discussion and address any tolerance shown by mainstream chiropractors and regulators. Thereafter, we discuss where the responsibility lies for stopping this type of misinformation.

## Main text

### Search

Over an 11-day period between 16 March and 26 March 2020, a group of 19 chiropractors searched social media and the internet for instances of chiropractors making claims related to immunity, chiropractic care and COVID-19. The material was sent around to colleagues, and the search snowballed from there, i.e. every post we came across led to a search to “dig further” for other types of misinformation. All in turn contacted their own professional networks with the intention of collecting as many different examples as possible in order to document which deleterious statements/claims were made concerning adjustments and the COVID-19 pandemic. Screenshots were taken in each case and the material was categorized according to claim and argument. Thus, we did not systematically search social media or the internet and kept no record of how many sites we examined.

Material from individual chiropractors and chiropractic clinics written in English, French, Swedish, Danish or Norwegian were gathered over a period of 11 days, after which saturation of statements and arguments was achieved. However, an extra statement was brought to our attention on April 10th. The examples provided herein are presented without identifying information, but screenshots and dates of capture are kept on record.

Thereafter, websites of all the Councils on Chiropractic Education (CCE), some CCE-accredited chiropractic teaching institutions, some chiropractic associations and regulatory boards were searched. These were assessed for unsubstantiated claims regarding COVID-19 and immunity boosting through adjustments. For convenience reasons, we limited this search to Europe, North America, South Africa, and Australia.

### Information from chiropractors

**Fig. 1:**
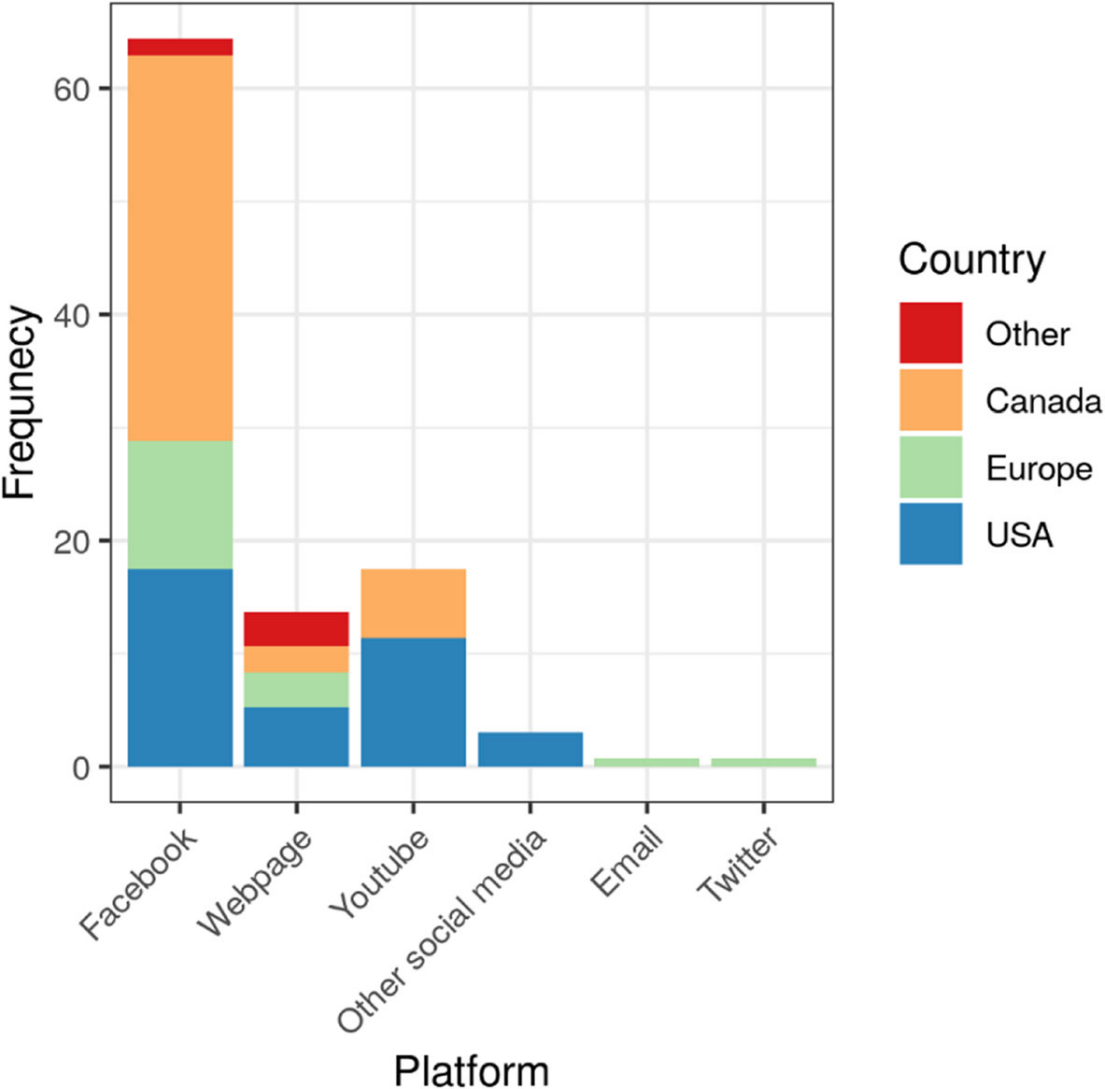
An overview of the social media and internet origins as well as the countries from where claims originated from chiropractors that adjustments are beneficial in the prevention of COVID-19

In this convenience sample, 99 relevant statements were identified from individual chiropractors and chiropractic practices, as posted on social media. An overview of the origins (platforms and countries) for these posts is presented in Fig. 1. However, this figure should not be construed as an estimate of prevalence, which is essentially unknown. It reflects the highly selective results of specific online searches and social media surfing in an eleven-day time period.

It should be noted, however, that the searches did not focus on general statements about COVID-19 but rather collected inappropriate or scientifically inaccurate statements about chiropractic, i.e. it’s supposed effect on immune function and, in particular, it’s supposed effect on COVID-19. Thus, all retained posts were in direct opposition to the WFC statement concerning COVID-19 [2]. Therefore, this work does not intend to provide information on the prevalence of messages that were opposed to the WHO guidelines [25]. Rather, it provides a series of cases of unsubstantiated claims.

**Fig. 2:**
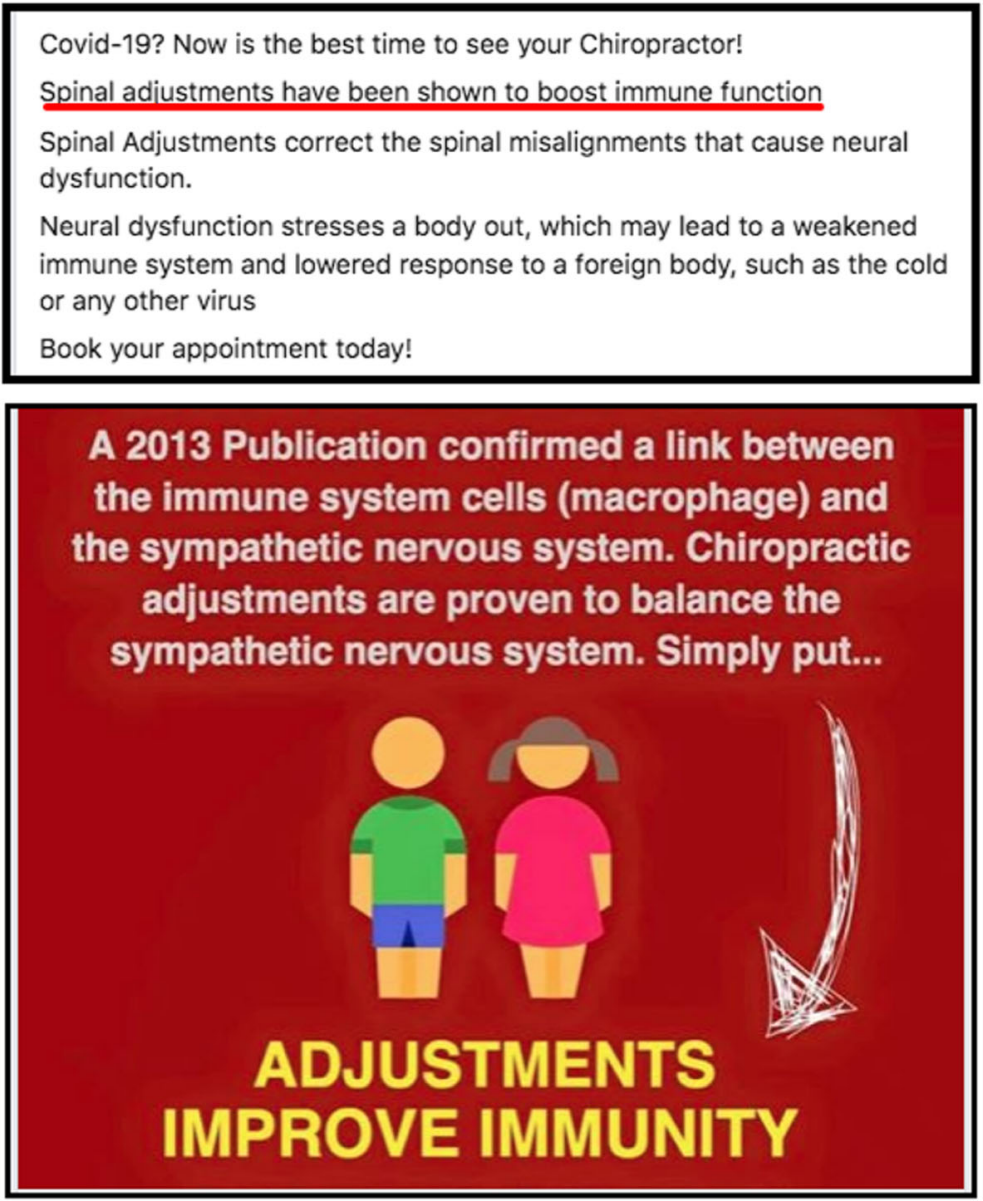
Examples that chiropractic adjustment will boost the immune system

The common element of the collected media statements is that adjustments boost the immune system. About half did not mention any public health intervention. However, the other half also mentioned factors such as hygiene, sleep, and stress management. Examples of such claims are provided in Fig. 2 [26].

**Fig. 3:**
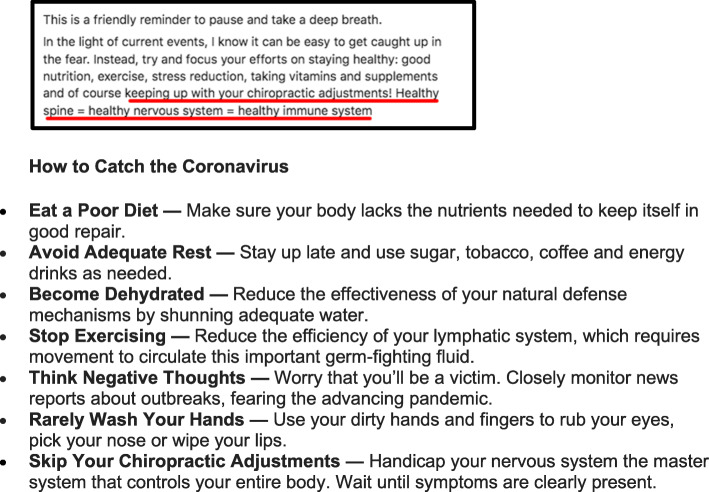
Examples of referring claims to old chiropractic concepts: a healthy immune system requires a healthy spine

Many claims (28% of the statements) could be traced directly to the more than 100-year old chiropractic concepts of the healthy spine and its purported effect on general health, including immune function (e.g. Figure 3) [27]. We are not aware of any scientific clinical evidence to support such beliefs.

**Fig. 4:**
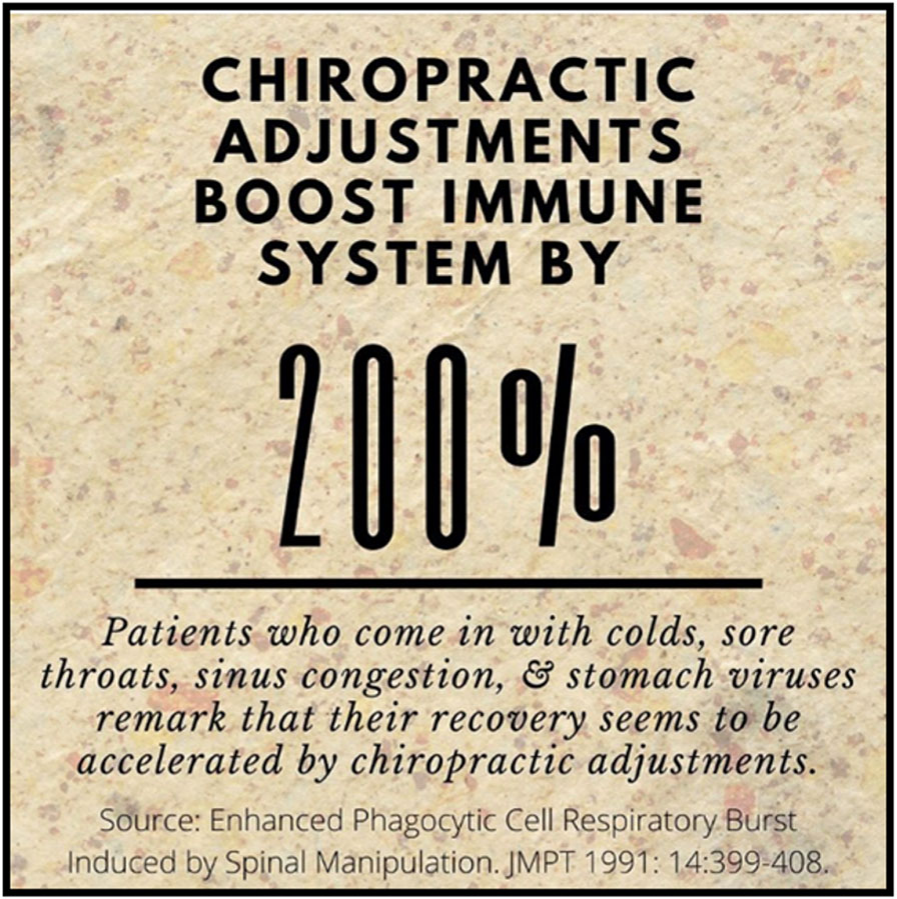
An example of referring claims that chiropractic adjustment boosts the immune system to a specific scientific paper

The type and quality of “evidence” provided to support these statements were not sufficient to validate the claims. Figure 4 [28] shows an example of an explicit reference to a specific scientific article. In this reference, the clinical meaning of the study’s results is not known, and, in fact, the article does not provide evidence that adjustments enhance or confer immunity, as previously summarized in a rapid review from the WFC [29].

**Fig. 5:**
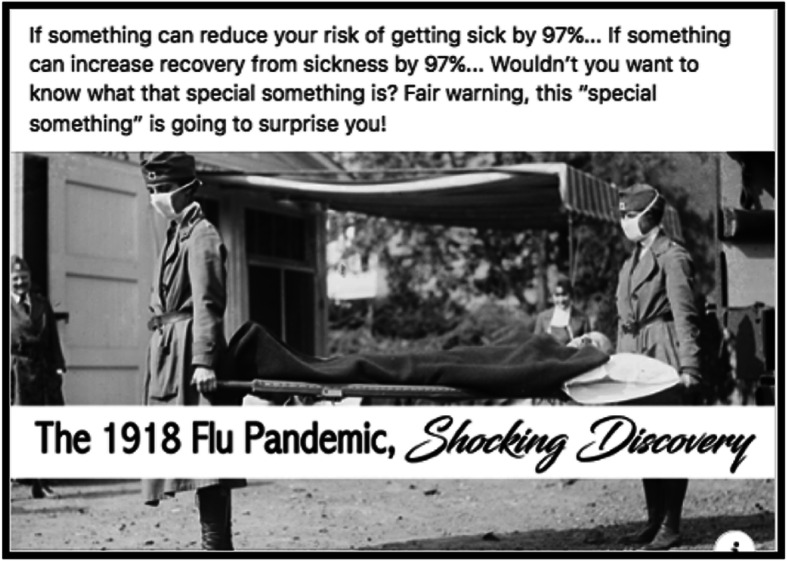
Example of claiming that people receiving chiropractic care during the 1918 pandemic were less likely to die

The most extraordinary claim that we found (17% of the statements that purported to be supported by evidence), was that mortality related to the Spanish flu epidemic after World War I was reduced among individuals who consulted chiropractors. According to the chiropractors who cited this study, patients treated with adjustments were less likely to die from the influenza than those treated by medical doctors. One example from a chiropractor’s website is shown in Fig. 5 [30]. However, the evidence for this claim seems to be an article from the January 1920 *Journal of the American Osteopathic Association*, reprinted in the May 2013 edition [31]. This paper claimed that patients with influenza or pneumonia attended by osteopaths died at 1/40th the rate of those attended by medical doctors. The mortality estimates for patients receiving medical care were collated using information obtained from 148 state and city health commissioners in the United States. Osteopathic mortality estimates were generated using self-reported data from 2445 members of the American Osteopathic Association. Anecdotal work with spurious comparison of blatantly incomparable groups would not be published in a modern reputable journal. Misquoting and misinterpreting this biased information to the public during a pandemic is irresponsible.

**Fig. 6:**
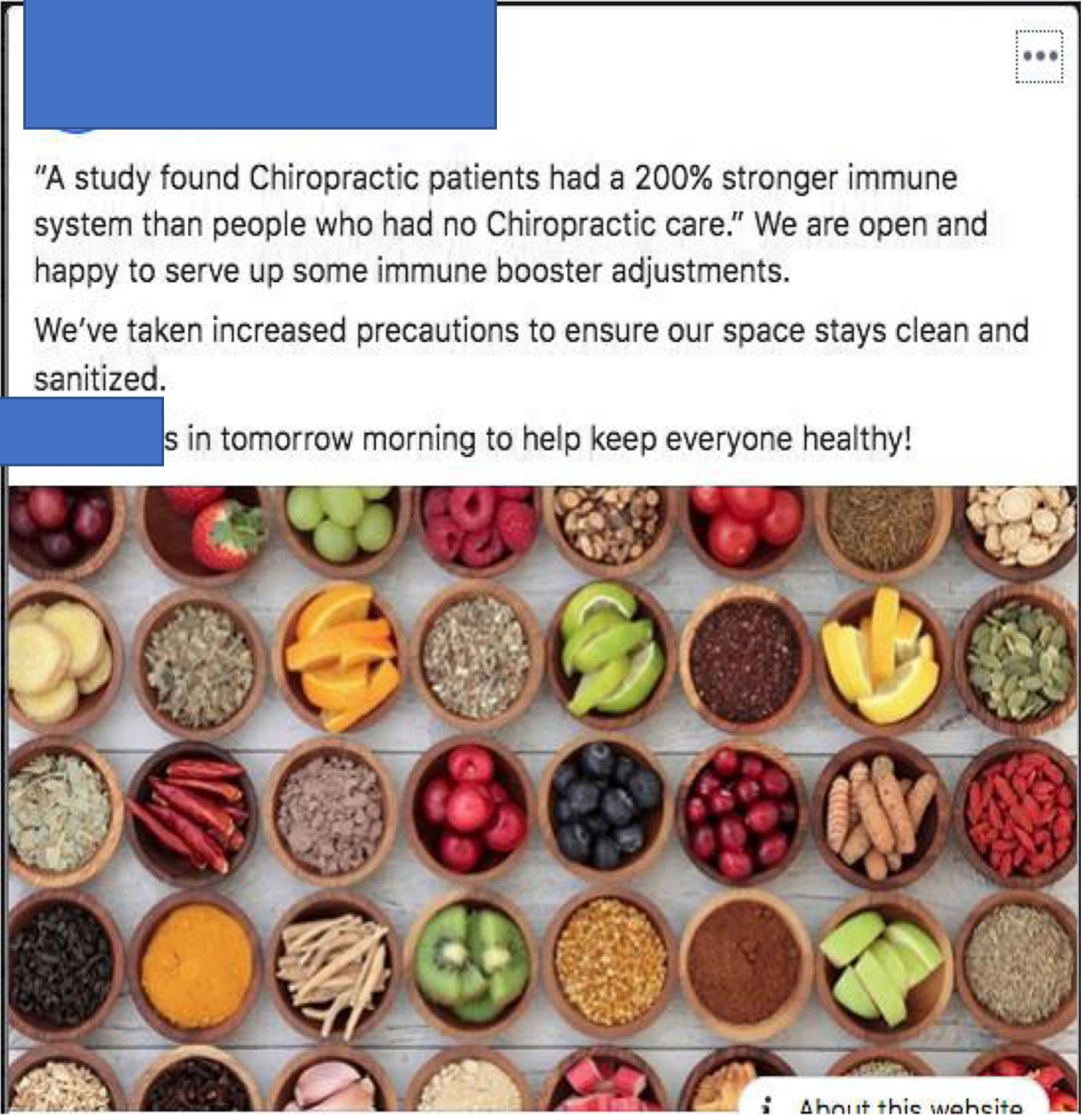
An example of referring to “evidence”

A common finding (29% of the statements “supported by evidence”) was that several claims referred to unspecified evidence by stating “research shows” or “evidence shows” (Fig. 6) [32]. To our knowledge no such generally acceptable and clinically relevant research exists to support such statements. It would be helpful if the chiropractors who make such claims would provide references for this anecdotal information.

One chiropractor boldly stated that “experience” shows that chiropractic treatment has a positive effect on the immune system. This was supported by patient testimonials. Testimonials are questioned as a marketing tool in many areas of medicine [20, 33, 34], as it is not possible to confirm such claims empirically and may lead readers to disregard valid health information [35]. Further, because of their propensity to mislead, testimonial use by health care providers, including chiropractors, contravenes advertising laws in some jurisdictions [20].

**Fig. 7:**
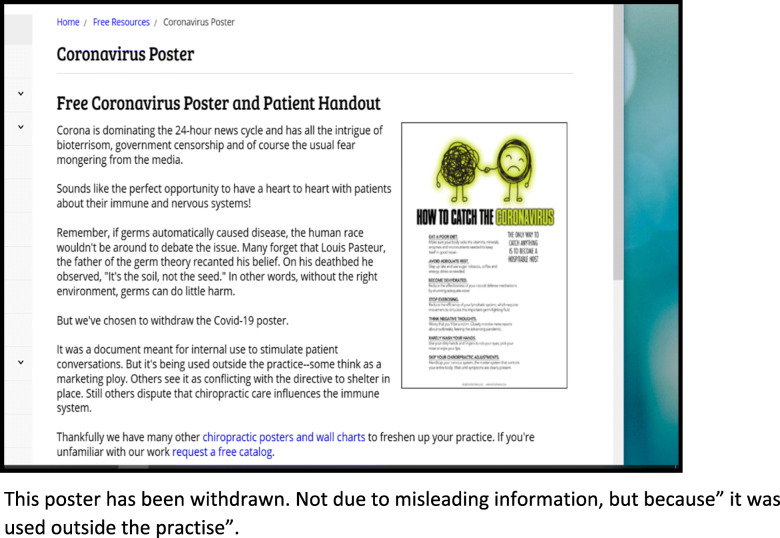
An example of claims stating: it is not about the germ, it’s about the immune system. This poster has been withdrawn. Not due to misleading information, but because” it was used outside the practise”

Other claims (4%) questioned undisputed and established science, such as the germ theory, and did not use “evidence” to back their claims. An example is shown in Fig. 7 [36].

**Fig. 8:**
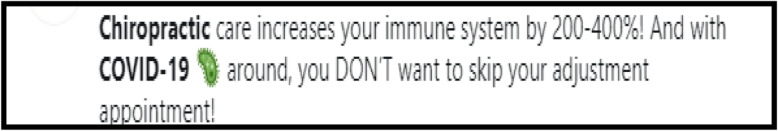
An example of claiming to boost immunity without any reference to evidence

A few (12%) of the statements were not accompanied by any argument at all as seen in Fig. 8 [37].

**Fig. 9:**
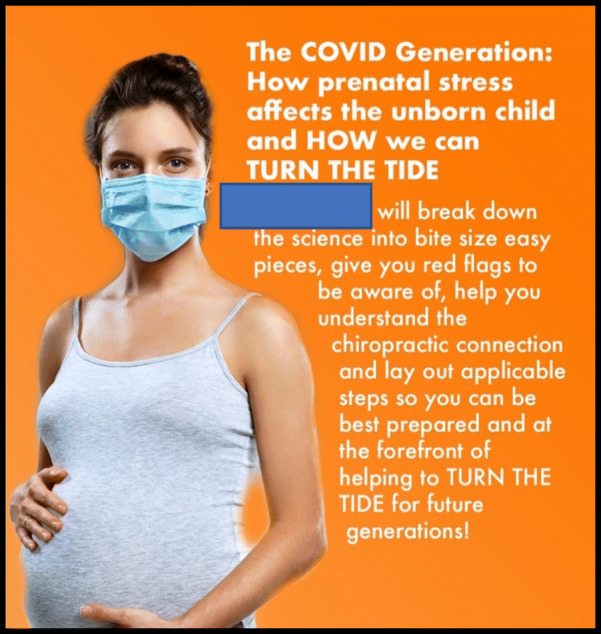
The claim that effects of COVID-19 stress on the unborn baby may be alleviated by chiropractic care

One statement concerned the risk to the unborn child, resulting from prenatal stress caused by COVID-19. A chiropractor claimed that pregnant women should get chiropractic care in order to minimize consequences on the baby (Fig. 9) [38]. To our knowledge, there is no compelling clinically relevant evidence to support such a statement.

### Information from chiropractic regulatory agencies

Information was collected from the websites of all chiropractic regulatory agencies in the US, Canada, the UK, New Zealand, and Australia. None advocated chiropractic care to improve immunity or to treat this viral disorder. Most had information from local health authorities concerning COVID-19, pertaining to washing hands, disinfecting equipment, and/or closing down practises. In Table 1, the statements are listed from the six regulators that specifically state that they support the WFC statement on chiropractic and immunity. The General Council on Chiropractic in the UK threatens regulatory action if chiropractors breach the requirements of the chiropractic code of practice. The regulatory boards of Minnesota and West Virginia both state that claiming chiropractic care is beneficial to prevent or treat COVID-19 is in violation of state statue pertaining to the scope of practice and advertising and the Chiropractic Board of Australia mentions unlawful advertising.

**Table 1:** A list of statements from 14 chiropractic regulatory boards in the UK, Australia, the US and Canada, which discourage the use of chiropractic care in the prevention and treatment for COVID-19 or to boost immunity in general

Regulatory board	Web-page	Statement
UK- General Council on Chiropractic	www.gcc-uk.org/	It has come to our attention that some chiropractors are claiming or implying in published material that spinal adjustment and/or manipulation may protect patients from contracting COVID-19, boosting the immune system or aiding recovery. The General Council on Chiropractic is clear that there is no credible scientific evidence that supports this and chiropractors must not make such a claim, or link treatment to COVID-19, in any way. Any chiropractors making such claims in any way, or making other unsubstantiated claims, run a very serious risk of being in breach of the requirements set out in the Code and regulatory action will be taken accordingly.
Chiropractic Board Australia	www.chiropracticboard.gov.au/	Other than sharing health information from authoritative sources, registered health practitioners should not make advertising claims on preventing or protecting patients and health consumers from contracting COVID-19 or accelerating recovery from COVID-19. To do so involves risk to public safety and may be unlawful advertising. For example, we are seeing some advertising claims that spinal adjustment/manipulation, acupuncture and some products confer or boost immunity or enhance recovery from COVID-19 when there is no acceptable evidence in support.
US- Arizona	https://chiroboard.az.gov/	Chiropractors should refrain from any communication that suggests spinal adjustment/manipulation may protect patients from contracting COVID-19 or will enhance their recovery. Doing otherwise is potentially dangerous to public health.
US- Minnesota	https://mn.gov/boards/chiropractic-examiners/	All licensees are advised that anyone making claims that adjustments can provide immunity to the flu or coronavirus is likely in violation of statute and rule relating to both scope of practice and advertising claims.
US- Texas	http://www.tbce.state.tx.us/	The Texas Board of Chiropractic Examiners agrees with and endorses the statements made by the World Federation of Chiropractic relating to chiropractic treatment and COVID-19
US- West Virginia	https://boc.wv.gov/	Any licensed DC who advertises or makes false, deceptive or misleading statements that he or she can cure the coronavirus is in violation of state statute pertaining to the scope of practice and advertising. Our scope does not provide disease-based treatment.
British Columbia Canada	https://www.chirobc.com/	The College of Chiropractors of British Columbia has become aware that some registrants are promoting treatment or supplements as a means to boost the immune system and may imply that this will prevent infection from the novel coronavirus (COVID-19). Any such claims made by registrants are inappropriate. When such claims are brought to the attention of the College of Chiropractors of British Columbia they will be forwarded immediately to the Inquiry Committee for investigation. As stated in part 9.5 of the Professional Conduct Handbook “, *The prevention and treatment of infectious disease is not within the scope of chiropractic practice*.”
Alberta Canada^a^	https://albertachiro.com/	It is not appropriate to suggest that anecdotal remedies or adjustments will treat or prevent illness. No marketing should be executed around COVID-19
Saskatchewan Canada^a^	https://saskchiro.ca/	Link to WFC statement
Ontario, Canada	https://www.cco.on.ca/	… with respect to inappropriate claims about COVID-19 made for chiropractic in social media and advertising. Where it was required, College of Chiropractors Ontario acted quickly and forcefully in reaching out to members whose posts may have crossed the line.
Quebec, Canada	https://www.ordredeschiropraticiens.ca/	We would like to remind everyone that the scope of chiropractic practice is the diagnosis, treatment and prevention of neuromusculoskeletal disorders. Any communication suggesting that chiropractic care can help people cope with the COVID-19 pandemic should be reported to the College.
New Brunswick, Canada^a^	https://nbchiropractic.ca/	Chiropractors should avoid making any unsubstantiated claims concerning the role of chiropractic care in preventing or managing COVID-19, or related viral infections. The New Brunswick Chiropractic Association will be monitoring social media and online content to ensure compliance with advertising standards. Failure to comply may lead to a complaint.
Nova Scotia, Canada	https://www.knowyourback.ca/	Private communications to registrants about not making false and misleading claims about chiropractic treatment and immunity regarding COVID-19.
Prince Edward Island, Canada^a^	https://www.peichiropractic.ca/	Members should avoid making any unsubstantiated claims concerning the role of chiropractic care in preventing or managing COVID-19, or related viral infections. The Prince Edward Island Chiropractic Association will be monitoring social media and online content to ensure compliance with advertising standards. Failure to comply may lead to a complaint.

^a^in some Canadian provinces, the professional association and the regulatory college exist as one entity

### Information from chiropractic teaching institutions

Information was collected from all the websites of CCE-accredited chiropractic teaching institutions in North America, Australia, New Zealand, South Africa, and Europe. At the time of data collection, most institutions had not posted any messages relating to advice on the pandemic. However, those which did, supported the WFC statement. Therefore, we do not further describe their contribution.

### Information from chiropractic associations

To explore what chiropractic associations disseminated in terms of information on the pandemic, we also searched the websites and Facebook of chiropractic associations from Europe (some), Australia (both), New Zealand (one), the US (all), and Canada (all), searching for information on the pandemic or the immune system in general.

Many national chiropractic associations clearly supported the WFC statement [2]: The American, Canadian, French, Norwegian and Swedish Chiropractic Associations, Chiropractic Australia, the General Chiropractic Council in Britain and the UK Chiropractic Council, as did the US associations in Illinois, Alabama, California, Kentucky, Minnesota, Missouri, New Mexico, Pennsylvania, and Texas.

### Councils on chiropractic education (CCEs)

A search of the web-pages for the four CCEs (responsible for Europe, Australia, Canada and the US, respectively) and the International umbrella organization (I-CCE), did not reveal the use of inappropriate information relating to chiropractic treatment and viral disorders.

### Main points

Some “traditional” chiropractors are making claims that could harm the public. In our opinion, the concept of adjustment- immunity boosting lacks biological plausibility when viewed through the lens of modern scientific knowledge.

There are of course situations where the scientific evidence is lacking and the biological plausibility is disputed, where high-quality randomized clinical trials of spinal manipulation may prove relevant. However, infectious disease is not one of them; the risks to the public are too high. Presently there is no generally accepted and clinically relevant evidence that adjustments protect against, nor impact, viral disease. Nevertheless, the “traditional” chiropractic approach, which forms the basis for the treatment to improve the immune system, survives among some chiropractors, despite scientific advancements over the last century. Some studies suggest that there has been a reluctance to denounce the “traditional” faction of the profession because the majority, middle-of-the-road chiropractors and some of their organisations purposely accommodate all perspectives under the title “chiropractic” and avoid internal conflict [39, 40].

The authors of this article, in line with modern-day concepts, assert that contemporary healthcare cannot be based on ideological beliefs and dogmatic contentions. When “traditional” chiropractors note the lack of evidence, we have observed that they often respond with a science type cliché: “absence of evidence is not evidence of absence”. This cannot be used as an excuse to discard biological principles, common sense, and critical appraisal. If claims are extraordinary and contrary to other generally accepted existing knowledge, the evidence provided must be especially convincing [41, 42].

### Responsibility for public safety is shared

Correcting misinformation is particularly important, because misinformation reaches more people and spreads more rapidly than accurate news [43]. This is especially true for health care misinformation [44], identified by the WHO as “infodemic” [45]. However, “traditional” chiropractors, who believe in and spread misinformation, seem particularly motivated to reject correction, especially if it poses a threat to important aspects of their cultural identity [46]. Therefore, it would be important to identify the origins of this problem in the chain of responsibilities, from educational institutions to individual clinicians, professional associations, and regulatory bodies.

In all professional groups there will be outliers, people who do not fit in with the mainstream. This is probably unavoidable. However, how likely is it that a substantial group of medical practitioners still believe in the practise of bloodletting and still reject the germ theory? In our opinion, we as a profession must take stronger measures to combat the dangerous anti-science sentiment that remains as a small but prominent part of the chiropractic community.

### The role of individuals

Chiropractors should reflect on their personal beliefs and what it means to be a health care professional, entrusted with ensuring public benefit from their services. Those who make unfounded claims breach a fiduciary agreement that allows for professional self-regulation and permits professionals a great deal of autonomy, provided they adhere to the duty of care, the duty of loyalty, and the duty of disclosure. For health care professionals, this means that they must put patient interests ahead of their own, stay up to date with current best practises, and fully inform patients of treatment options, including risks/benefits of any given procedure. Chiropractors who put their personal beliefs ahead of evidence or who selectively choose evidence that supports their belief system fail to reach these standards and breach their professional duties, are legally negligent, and do not act in the best interests of their patients.

### The role of teaching institutions

We would argue that the chiropractic teaching institutions are obligated to set the example for best practice by taking public stances on issues such as the present COVID-19 crisis. Teaching institutions, with their staff of highly educated and clinically experienced experts, are sought out for guidance, particularly at times of controversy and crisis [47, 48]. Therefore, they bear a responsibility to act as prominent advocates of science and reason and must emphasize the teaching of evidence-based care and de-emphasize historical beliefs. Most of the institutions that posted information on the COVID-19 pandemic did so in a responsible manner.

### The role of accrediting agencies

Four CCEs are tasked with accrediting chiropractic programs within their respective geographical region. However, the standards and processes of these CCEs are not internationally homogeneous and concerns have been raised about some accredited chiropractic programs and the subsequent practice patterns demonstrated by their graduates [12, 13, 30–33]. Further, some CCEs have created a” big tent” approach to their accreditation standards and processes, resulting in all types of understanding of the term chiropractic [21, 22]. This approach can make it difficult to define the practice and scope of chiropractic [34]. It has been argued that some CCEs have failed to wholeheartedly embrace an evidence-based approach to education and practice [35–37]. For example, only 35% of students in some chiropractic programs agree that immunization is an effective disease prevention and 9% believe that SMT is an effective primary treatment for AIDS [49]. Further, many chiropractic students internationally have difficulty identifying non-indications for SMT [50, 51].

The COVID-19 crisis highlights the need for all CCEs to be agile in times of crisis and adopt the standards of mainstream healthcare and place patient welfare at the forefront of their agenda.

### The role of regulatory bodies

The groups entrusted with ensuring public safety must take steps to prevent pseudoscientific claims by chiropractors. A recent meta-analytic study suggested the following procedure for correcting misinformation [52]. First, make known the poor credibility of the source of the misinformation. Second, corrections should come from those who created and distributed the misinformation, be made as soon as possible, and come from a credible source [53].

## Conclusions

In this search of public media in Europe, North America, New Zealand, and Australia, we discovered many cases of misinformation. Claims of chiropractic treatment improving immunity conflict with the advice from authorities and the scientific consensus. The science referenced by these claims is missing, flawed or has no clinical relevance. Consequently, their claims about clinical effectiveness are spurious at best and misleading at worst.

However, our examples cannot be used to make statements about the magnitude of the problem among practitioners as our samples were not intended to be representative. For that reason, we also did not include an analysis of the arguments provided in the various postings. In view of the seriousness of the topic, it would be relevant to conduct a systematic study on a representative sample of public statements, to better understand these issues.

Our search illustrates the possible danger to public health of misinformation posted on social media and the internet. This situation provides an opportunity for growth and maturation for the chiropractic profession. We hope that individual chiropractors will reflect on and improve their communication and practices. Further, we hope that the chiropractic teaching institutions, regulators, and professional organisations will always demonstrate responsible leadership in their respective domains by acting to ensure that all chiropractors understand and uphold their fiduciary duties.

## Data Availability

Examples of misinformation available from the authors upon reasonable request. The editor-in-chief has all the URL’s of the examples provided herein.

## Abbreviations

WHO: World Health Organisation
COVID-19: Severe Acute Respiratory Syndrome Coronavirus 2 (SARS-CoV-2)
SMT: Spinal Manipulative Therapy
WFC: World Federation of Chiropractic
CCE: Council on Chiropractic Education
US: The United States (of America)
UK: The United Kingdom
DC: Doctor of Chiropractic
